# The impact of monitoring techniques on progression to chronic breast cancer-related lymphedema: a meta-analysis comparing bioimpedance spectroscopy versus circumferential measurements

**DOI:** 10.1007/s10549-020-05988-6

**Published:** 2020-11-27

**Authors:** Chirag Shah, April Zambelli-Weiner, Nicole Delgado, Ashley Sier, Robert Bauserman, Jerrod Nelms

**Affiliations:** 1 Department of Radiation Oncology, Taussig Cancer Institute, Cleveland Clinic, Cleveland, OH USA; 2 TTi Health Research and Economics, Westminster, MD USA

**Keywords:** Breast cancer, Lymphedema, Axillary dissection, Bioimpedance

## Abstract

**Background:**

Chronic breast cancer-related lymphedema (BCRL) is a potentially serious complication following treatment. Monitoring for progression to BCRL may allow for earlier detection and intervention, reducing the rate of progression to chronic BCRL. Therefore, the purpose of this meta-analysis is to evaluate the impact of monitoring techniques on the incidence of chronic BCRL among patients monitored by bioimpedance spectroscopy (BIS) and circumference as compared to background rates.

**Methods:**

Eligible peer-reviewed studies from PubMed, CINHAL, or Google Scholar that were published in English from 2013 onward and conducted in North America, Europe, or Oceania. Incidence rates abstracted from studies were classified by BCRL monitoring method: background (no standardized BIS or circumference assessments), BIS or circumference. A random-effects model was used to calculate a pooled annualized estimate of BCRL incidence while accounting for clinical and methodological heterogeneity. Subgroup analyses examined differences in duration of follow-up as well as breast and axillary surgery.

**Results:**

50 studies were included, representing over 67,000 women. The annualized incidence of BCRL was 4.9% (95% CI: 4.3–5.5) for background studies (*n* = 35), 1.5% (95% CI: 0.6–2.4) for BIS-monitored studies (*n* = 7), and 7.7% (95% CI: 5.6–9.8) for circumference-monitored studies (*n* = 11). The cumulative BCRL incidence rate in BIS-monitored patients was 3.1% as compared to 12.9% with background monitoring (69% reduction) and 17.0% with circumference-monitored patients (81% reduction).

**Conclusions:**

Evidence suggests that monitoring with BIS allowing for early intervention significantly reduces the relative risk of chronic BCRL with a 69% and 81% reduction compared to background and circumference, respectively. Circumference monitoring did not appear to provide a benefit with respect to chronic BCRL incidence. Based on these results, BIS should be considered for BCRL screening in order to detect subclinical BCRL and reduce rates of chronic BCRL, particularly in high-risk patients.

**Electronic supplementary material:**

The online version of this article (10.1007/s10549-020-05988-6) contains supplementary material, which is available to authorized users.

## Introduction

Lymphedema is a common and potentially serious complication of breast cancer (BC) treatment and has been associated with the extent of breast and axillary surgery, regional nodal irradiation (RNI), and taxane-based chemotherapy. [1–3] A recent meta-analysis reported that breast cancer-related lymphedema (BCRL) affects approximately 19% of breast cancer patients with follow-up ≥ 12 and < 24 months after treatment. Significant heterogeneity in BCRL incidence rates exists due to different monitoring methods, follow-up periods, patient BCRL risk profiles, and study designs. [1, 4] Patients with chronic BCRL have reduced quality of life which can be attributed to functional impairment or disability, discomfort, and infection, as well as higher medical costs. [1,^ 2^,^ 5^–7] Psychologically, chronic BCRL may also cause anxiety, depression, and social difficulty. [1,^ 2^, 4] It is therefore important to identify BCRL early, in the subclinical phase of the process, allowing for earlier intervention, thereby reducing the development of the severe and irreversible symptoms of chronic BCRL. [5] In particular, patients who undergo mastectomy, axillary lymph node dissection (ALND), RNI, or taxane-based chemotherapy have an increased risk of developing BCRL and with being high-risk would benefit from prospective BCRL assessment. [8]

There are several different methods commonly used to detect for and monitor BCRL including tape measurement and water displacement [5–7, 9]. Both of these methods are considered standard for lymphedema monitoring. [7] However, these methods can be highly variable and prone to bias from human measurement, resulting in inter- and intra-observer variability, while being less able to detect subclinical BCRL. [7, 10] Bioelectrical impedance (bioimpedance) or bioimpedance spectroscopy (BIS) has emerged as a promising diagnostic tool, able to objectively quantify BCRL by measuring resistance to electrical current flow through a patient’s body, giving a more accurate and consistent representation of body composition, and aiding in the assessment of subclinical BCRL earlier than standard methods. [5,^ 6^,^ 8^, 9] Due to the capability of BIS to aid in the assessment of subclinical lymphedema and trigger early intervention, it can potentially reduce the rate of progression to chronic BCRL (clinically assessed BCRL occurring > 3 months postoperatively), resulting in substantial cost savings and improved quality of life, especially in high-risk patients. [3, 9, 11] However, because BIS has not been considered the standard BCRL monitoring method, it is not usually covered by health insurance companies despite studies demonstrating reduced chronic BCRL using BIS as well as cost-effectiveness with the technique. [5, 12].

A previous meta-analysis from DiSipio et al. evaluated the incidence of BCRL and risk factors finding that approximately one in five women will develop BCRL post-treatment with increased risk for those undergoing ALND and mastectomy [4]. We therefore conducted a meta-analysis in order to determine the relative reduction in progression to chronic BCRL among BC patients, comparing (a) BIS monitoring as a trigger for early intervention, using the L-Dex U400 device; (b) limb circumference monitoring with tape measure; and (c) the background rate of progression. The primary outcome of interest was annual progression to chronic BCRL in patients experiencing either (a) the background rate of progression, with either no monitoring or monitoring without standardized BCRL circumference or BIS assessments; (b) monitoring with BIS and early intervention; or (c) monitoring with tape measures of limb circumference with or without intervention.

## Materials and methods

### Eligibility criteria

With the intent to provide an update to the previous meta-analysis conducted by DiSipio et al. in 2013, we conducted a systematic literature review to identify studies investigating chronic BCRL occurrence or progression in adult women with breast cancer. [4] In order to be eligible, each peer-reviewed study must have been published in English in 2013 or later (up to 2019 when search was performed) and conducted in North America, Europe, or Oceania. Studies were included as background studies if they reported the BCRL rate over any postsurgical time frame in any group of female breast cancer patients, regardless of study design, as the previous meta-analysis did not exclude studies based on monitoring method. Prospective surveillance and retrospective chart review studies were included as circumference or BIS studies if they specifically described using the L-Dex U400 BIS device or the tape measure method, included a presurgical baseline measurement, and reported the BCRL rate over any postoperative time frame. Randomized controlled trials were included in the tape measure or BIS groups if they included a BIS group, a circumference-monitoring group, or both, with a presurgical baseline measurement, in direct comparison to each other or to a control group with no monitoring. All data abstraction, analyses, and interpretation were conducted without the involvement of the sponsor.

### Information sources and search strategy

The systematic literature search was conducted following PRISMA guidelines and focused on the electronic databases PUBMED and CINAHL. [13] In addition, a Google Scholar search was conducted in which the first three pages of results were reviewed to identify potential additional references. Reviewers also screened references cited in recent publications in which the L-Dex U400 device was used to identify additional studies potentially eligible for inclusion.

The literature search made use of the following terms to identify relevant publications without limiting by date: “breast cancer” AND (lymphedema OR lymphoedema OR “lymphatic edema”) AND (incidence OR prevalence OR rate); “breast cancer” AND (lymphedema OR lymphoedema OR “lymphatic edema”) AND (monitoring OR surveillance); or “breast cancer” AND (lymphedema OR lymphoedema OR “lymphatic edema”) AND (“bioelectrical impedance” OR “bioimpedance” OR “bioimpedance spectroscopy” OR “BIS” OR “L-Dex” OR “bioimpedance analysis” OR “BIA”).

### Reference screening

Two independent reviewers initially screened all study abstracts considered eligible. Exclusions during this stage were based on deviations of methodologies and populations from the predefined criteria, as well as sources other than peer-reviewed journals, and any duplicate references. The reviewers then compared their inclusion decisions for all abstracts. Any discrepancies were resolved through discussion; if a conclusion could not be reached, the decision for inclusion or exclusion was made by a senior author. A reference manager tool, Mendeley, allowed for compilation, screening, and tagging for inclusion/exclusion of the publications was identified from the systematic literature search. At this point, articles published prior to 2013 were excluded so the results would be a true extension of the DiSipio meta-analysis. [4] Next, the two reviewers conducted an independent screening of 10% of the full-length texts for each study and ensured there was 80% agreement in their decisions. [14] The each reviewer then reviewed half of the remaining full-text articles. This screening excluded any articles that a full-text reading found to lack BCRL incidence or progression rates, usable counts to determine rates, clearly defined follow-up periods, or identification of the specific device used if a BIS device was part of the methodology, or that included only self-reported lymphedema.

### Data abstraction

Following completion of the full-text review, two reviewers abstracted all elements of interest from the full-length texts. Primary abstraction of articles was performed by ND, with support by AS. All data abstracted were thoroughly checked by the other reviewer to ensure accuracy in abstraction. Any discrepancies throughout the abstraction process were resolved through discussion; if a conclusion could not be reached, decisions were made by a senior author. In addition to basic study information (year of publication, location, design, start and end dates for data collection, study duration, patient inclusion and exclusion criteria, percent of participants lost to follow-up, definition of progression to lymphedema, and lymphedema measurement methods), key data elements for each arm related to patient demographics, treatment, and development of lymphedema were abstracted. These included age, race, and body mass index (BMI) of participants; percent of patients experiencing mastectomy, breast conservation techniques, ALND, sentinel lymph node biopsy (SLNB), taxane chemotherapy, or RNI; the final number of patients in the study arm diagnosed with progression to chronic BCRL (in the case of studies with early intervention (which could be variable interventions including compression sleeve or other therapies), this meant the number diagnosed following intervention, not the number triggering early intervention); and the mean time to progression. To be included in the BIS monitoring group, the studies classified patients with chronic BCRL as having an L-Dex score of ten or greater; to be included in the circumference monitoring group, the studies used the accepted standard of two cm difference or more. If the studies used non-standard methods, such as volume calculations, they were included within the background group and categorized as monitoring. Studies could contribute data to more than one group if they included multiple arms and separate arms met inclusion criteria for different groups. Monitoring via bioimpedance assessment (BIA) is based on a smaller range of frequencies (or even a single frequency) than BIS and was assigned to the background group.

### Statistical analysis and synthesis

Stata (version 13; StataCorp LLC, College Station, Texas) was used to conduct all analyses. Synthesis of studies reporting rates of chronic BCRL in the absence of monitoring via tape measurement of limb circumference or BIS provided estimates for the background rate of chronic BCRL. Rate of progression to chronic BCRL was then synthesized for studies involving surveillance and early intervention based on BIS or tape measurement. A random-effects model was used in the analysis to account for both clinical and methodological heterogeneity. Known risk factors for BCRL were assessed via subgroup analyses. Sufficient data were available on the proportion of patients undergoing ALND, SLNB, and Mastectomy. Thresholds were selected empirically from the data. Other known risk factors not included due to a lack of data were BMI, taxane chemotherapy, and RNI.

Results are reported without transformation but were square-root transformed to help stabilize variability and confirm that heterogeneity was better controlled. Rates of progression to chronic BCRL were annualized from the data reported by each study, based on the length of reported follow-up. BCRL rates associated with BIS monitoring and with tape measurement were then compared to each other and to background rates. Heterogeneity for all estimates was acceptable (I [2] ≤ 50%) when data were square-root transformed, with a few exceptions (Tables 3, 4). In Table 3, the exceptions were overall pooled estimate for circumference-monitored studies (I^2^ = 61·0%); circumference-monitored studies for > 2 years (I^2^ = 82·6%); circumference-monitored, prospective studies (I^2^ = 74·6%); and background RCT studies (I^2^ = 61·3%). In Table 4, the exceptions were circumference-monitored for ALND > 50% (I^2=^55·9%), circumference-monitored & both SLNB analyses (SNLB ≤ 50%: I^2^ = 85·0%; SLNB > 50: I^2^ = 54·2%), background rate for ALND > 50% (I^2^ = 57·5%), and background rate for SLNB ≤ 50% (I^2^ = 65·3%).

## Results

**Fig. 1:**
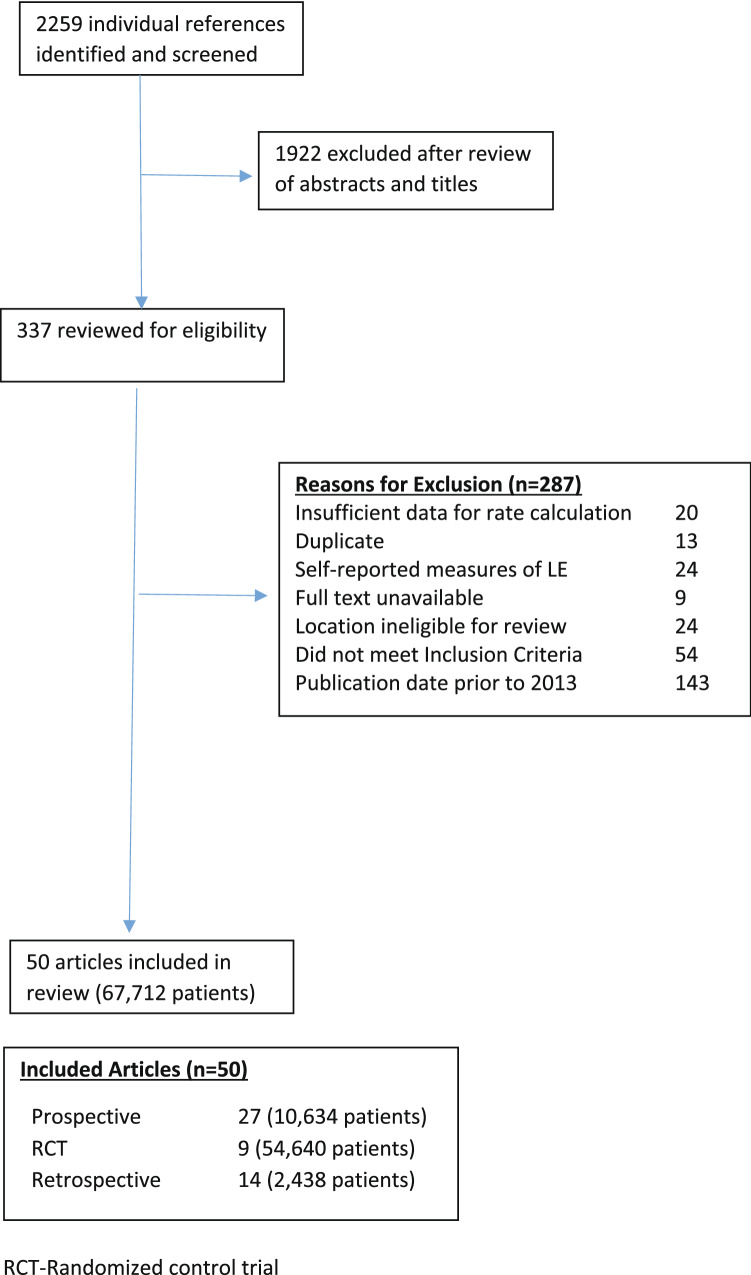
CONSORT Diagram

A total of 2,259 individual references were identified from the literature searches (Fig. 1). Of these, 1922 (85·1%) were excluded during the initial review of abstracts and titles for not meeting eligibility criteria. Of the remaining 337 studies which then underwent full-text review, 143 studies were excluded due to being published prior to 2013. Of the remaining 194, an additional 144 articles (74·2%) were excluded, leaving 50 articles included in the analysis. Figure 1 details reasons for exclusion based on full-text review. Of the 50 studies remaining for inclusion in the meta-analysis, 27 were prospective surveillance designs, 14 were retrospective chart reviews, and nine were RCTs. 35 studies provided estimates of the background rate of progression to BCRL; 11 studies provided estimates for monitoring with tape measurement; and 7 studies provided estimates for monitoring with BIS. Three studies contributed data to more than one arm. [15–17] One study reported an incidence of 0, which was set to 0·5. This continuity correction is supported in the literature for analyses including only one study with a zero count. [18, 19] Mean or median length of follow-up across studies ranged from one to ten years. Tables 1 and 2 and Supplemental Table S1 summarize characteristics of the studies included; of note differences in the rates of ALND did exist. [8, 15–17, 20–65].

**Table 1:** Patient population characteristics

Study type	Studies included in analysis	Total patients (*n*)	Mastectomy (%)	Breast conservation (%)	ALND (%)	SLNB (%)	Taxane chemotherapy (%)	RNI (%)
All Background Studies	35	57,944	20,472 (35%)	36,631 (63%)	16,888 (29%)	30,754 (53%)	1,502 of 5,398 patients (27%)^b^	2,607 of 10,184 patients (25%)^b^
Background (no monitoring studies only)	11	48,833	18,392 (37%)	30,246 (61%)	12,649 (25%)	26,058 (53%)	533 of 2,216 patients (24%)^b^	503 of 2,680 patients (18%)^b^
BIS Studies	7	1,924	623 of 1,165 patients (53%)^b^	541 of 1,165 patients (46%)†	547 (28%)	1125 (58%)	314 of 948 patients (33%)^b^	182 of 985 patients (18%)^b^
Circumference Studies	11	8,403	5,362 of 5,843 patients (91%)^b^	736 (8%)	6,213 (73%)	2019 (24%)	32 of 71 patients (46%)^b^	139 of 180 patients (77%)^b^
All Types^a^	50	67,712	26,436 (39%)	37,858 (55%)	23,134 (34%)	33,845 (49%)	1,817 of 6,383 patients (28%)^b^	2,219 of 12,217 patients (18%)^b^

^a^Total number of patients across all study arms. Please note that Blaney, Armer, and Ridner have patients contributing to multiple arms

^b^Very few studies contributed. Used only the total number of patients for contributing

Note: *ALND* Axillary Lymph Node Dissection, *SLNB* Sentinel Lymph Node Biopsy, *RNI* Regional Node Irradiation

**Table 2:** Study characteristics

Study	Location	Inclusion criteria	Exclusion criteria	Total patients (n)	Duration of follow-up (mean/median), years	LE definition	Patients progressed to BCRL (n)	Annual rate of progression (patients progressed/person-time)
Ammitzboll et al. [20]	Denmark	Women aged 18–75, operated with ALND for unilateral primary BC, able transport themselves, and able to participate in exercise intervention	Previous ALND either side, primary breast reconstruction, metastatic disease, and history of lymphoedema	6	0·96	> 3% interlimb volume difference (ILVD), increased score on the numeric rating scale (NRS) for cardinal symptoms of swelling, and if meeting two of four clinical assessment criteria	1	0·17
Ammitzboll et al. [21]	Denmark	Women age 18–75 years, primary unilateral BC, ALND, and ability to participate in group exercise regimen	Distant metastases, previous ALND on contralateral side, and history of arm lymphedema	158	1·00	> 3% increase in ILVD	32	0·20
Armer et al. [15]	USA	Women over 18, with cT0-T4, NI-2, M0 breast cancer, fine needle aspiration or core needle biopsy of axillary node documenting nodal metastasis at diagnosis	Prior ipsilateral axillary surgery, or prior SLNB; patients who had bilateral breast cancer, current limb infection, lymphangitis, or any other condition that would affect testing	488	3·00	A volume increase > 10% compared to baseline and /or the contralateral arm	294	0·20
Bains et al. [23]	United Kingdom	Invasive breast cancer and undergoing axillary lymph node surgery	Not specified	38	1·12	5–10% volume increase from Perometer and clinical signs of BCRL	7	0·17
Barrio et al. [25]	USA	Female patients age > 18, newly diagnosed invasive or in situ breast carcinoma, and planned unilateral axillary surgery with either SLNB or ALND	Not specified	186	1·52	Relative arm volume difference (RAVD) of > 10%	13	0·05
Basta et al. [26]	USA	Patients with breast cancer diagnosis and mastectomy	Patients not meeting mastectomy requirement, having lymphedema before mastectomy, and < 12 months of follow-up or mortality within 12 months of mastectomy	3,136	4·20	ICD-9 DX codes 457·0 and 457·1	325	0·02
Berlit et al. [28]	Germany	Used study sample from previously conducted study	Not specified	42	1·00	N/A – used circumference measurements and defined in previous study	7	0·17
Black et al. [29]	USA	Pathologically node-negative disease and a documented axillary surgical procedure on women aged 66 years or older	Not specified	31,274	5·00	ICD-9 DX codes 457·0 and 457·1 for Lymphedema	2502	0·08
Blaney et al. (2014) [16]	Ireland	Women, aged 18–99 years, newly diagnosed with stages I–III unilateral breast cancer from specialist diagnostic breast clinics in Belfast City Hospital and Ulster Hospital	History of breast cancer or lymphoedema, prior severe trauma/surgery upper limb, pacemaker/ metal implant, no baseline assessment, Stage IV disease, and other additional conditions (see Appendix)	71	1·00	BIA classification: L-Dex score of > 10 or a 10 U increase from baseline	31	0·44
Bloomquist et al. [30]	Denmark	Breast cancer patients from exercise intervention, had received ≥ 1 cycle of chemotherapy, had a WHO performance status of 0 or 1 and approved to participate by treating oncologist	Patients that had recurrent cancer, deceased, or diagnosed BCRL prior to participation in "Body & Cancer"	149	1·17	An interlimb difference of > 2 cm at to two or more measures	41	0·24
Boccardo et al. [31]	Italy	Candidates for Lymphatic Microsurgical Preventing Healing Approach (LYMPHA) procedure during ALND	No afferent lymphatics could be found and because of massive metastatic disease	74	4·00	Missing–not specified. Volumetry used	3	0·01
Bulley et al. [32]	United Kingdom	Women who had completed treatment (surgery, chemotherapy and radiotherapy), and who were attending review appointments	If they had recurrence, or were unable to complete non-translated questionnaires	613	5·25	Limb volume difference (LVD) was 10% or greater (unilateral treatment only)	115	0·04
Cariati et al. [33]	England	All patients diagnosed with breast cancer who underwent ALND	Not specified	273	2·67	> 10% arm volume increase compared to contralateral arm by perometer or subjective assessment	74	0·10
Cintolesi et al. [34]	United Kingdom	Women newly diagnosed with breast cancer	Not specified	26	2·08	Clinical Assessment Criteria or by the volume of each upper limb changes from baseline by Perometer	10	0·18
Coromilas et al. [35]	USA	From SEER-Medicare database, women aged 65–90 with DCIS who underwent excisional breast procedure (BCS), simple mastectomy, total mastectomy with ALND, or skin-sparing mastectomy	Excluded patients with any code for invasive breast cancer	10,504	1·00	ICD-9 DX codes 457·0 and 457·1	714	0·07
Cowher et al. [36]	USA	Women undergoing mastectomy with CARE performed by the same surgeon	Not specified	587	5·10	Occupational therapist or surgeon’s note documenting a decreased function and/or quality of life due to arm swelling or pain	20	0·01
De Groef et al. [38]	Belgium	Women treated for breast cancer, radiation therapy terminated ≥ 3 months ago, upper limb region pain score of ≥ 40 of 100 on VAS, presence of myofascial dysfunctions at upper limb region	Unable to attend therapeutic sessions and measurements for the entire duration, presence of shoulder pathologies for which surgical indications exist, or presence of current episodes of cancer or metastasis	50	1·00	> 5·0% increase of relative arm volume difference compared to baseline	23	0·46
Feldman et al. [40]	USA	Female patients with breast cancer and documented axillary nodal metastasis undergoing planned ALND or modified radical mastectomy	Those not undergoing complete ALND, allergy to Lymphazurin blue dye, and pregnancy	37	0·50	> 2 cm discrepancy in circumferential size measurements between the affected and unaffected arms or a change from baseline	7	0·38
Fu et al. [42]	USA	Women over 21 yoa, first time diagnosis of breast cancer (Stage I- III), and scheduled for surgical treatment	Women with metastatic cancer (Stage IV), prior history of breast cancer and lymphedema, and bilateral breast cancer	140	1·00	10% LV increase from baseline in the ipsilateral arm compared to contralateral arm by perometer	4	0·03
Fu et al. [43]	USA	Patients with non-metastatic invasive breast cancer who had undergone mastectomy and either SLNB or ALND as their first nodal surgery	Under 18, lumpectomy, no nodal surgery, pN stage: N0, neoadjuvant chemotherapy, cancer within 5 years	151	3·63	Not specified	28	0·05
Fu et al. [41]	USA	Women over 21 yoa, first time diagnosis of breast cancer (stages 1–3), scheduled for surgical treatment	Diagnosed with metastatic cancer, prior history of breast cancer and lymphedema, bilateral breast cancer, artificial knee or hip, kidney failure, heart failure	136	1·00	L-Dex ratios greater than 7·1.[BIA measurements]	18	0·13
Gennaro et al. [44]	Italy	Consecutive patients undergoing ARM with a diagnosis of axillary nodal involvement based on preoperative needle biopsy or a positive SLNB and scheduled for ALND	Not specified	60	1·33	Arm circumference measurements increasing by > 2 cm, and/or based on subjective symptoms and/or comparative lymphoscintigraphy	9	0·11
Hopkins et al. [45]	USA	321 of the CALGB 49,907 patients were also enrolled in a QOL companion trial	Not specified	321	2·00	Physician reported BCRL; Progression not specified	24	0·04
Jammallo et al. [47]	USA	Patients undergoing treatment for primary breast underwent prospective lymphedema screening, had preoperative and postoperative measurements by Perometer	Previous bilateral breast surgery and measurements occurring after a patient was diagnosed with metastatic disease	787	2·25	RVC > 10% occurring > 3 months postoperative	22	0·01
Jammallo et al. [46]	USA	Women undergoing treatment for newly diagnosed breast	Previous bilateral breast surgery and those with metastatic disease were excluded from the analysis	324	1·25	RVC greater than or equal to 10%	20	0·05
Koehler et al. [49]	USA	Women having undergone early-stage surgical breast cancer treatment (lumpectomy or mastectomy) with a minimum removal of one axillary lymph node by SLNB	Previous surgical treatment for breast cancer or synchronous bilateral breast cancer, or a previous history of shoulder surgery, shoulder dysfunction, or history of upper extremity deep vein thrombosis	36	1·50	> 10% difference in volume, > 200 mL difference in volume, > 2 cm difference at any site on the upper extremity using circumferential measurements or an L-Dex > 10	6	0·11
Nguyen et al. [53]	USA	All incident breast cancer cases diagnosed in Olmsted County, MN residents in the Olmsted County Rochester Epidemiol-ogy Project Breast Cancer Cohort	Not specified	1,794	10·00	All cases with definite or probable lymphedema in the clinical notes	209	0·01
O’Toole et al. [55]	USA	Women with primary breast cancer, underwent unilateral breast surgery, underwent preoperative and postoperative assessments, and at least one follow-up assessment > 3 months after surgery	Patients with bilateral breast or axillary surgery, distant metastasis or recurrence	308	1·36	An arm volume increase of > 5% RVC	50	0·12
Sagen et al. [56]	Norway	ages 35–75 years, diagnosed with early-stage primary breast cancer, surgery with breast ablation or breast-conserving surgery with ALND or SLNB only	Too frail, inability to cognitively comprehend, metastasized breast cancer or other cancer, preoperative injuries, pathologic problems affecting upper limb function while performing tests	391	2·50	10% arm volume increase compared to contralateral arm by Perometer	40	0·04
Skuli et al. [57]	USA	Patient cohort who participated in a 60- to 90-min SV after referral by patients’ medical oncologists and occurred 1–3 months after completion of locoregional therapy and initial systemic therapy	Not specified	87	2·42	N/A–retrospective chart review	20	0·10
Specht et al. [59]	USA	Women diagnosed with breast cancer	Patients who underwent bilateral breast or axillary surgery were excluded	1,173	4·00	RVC > 10% measured at least 3 months after surgery	114	0·02
Swaroop et al. [60]	USA	Women diagnosed with unilateral breast cancer who underwent surgery and prospective screening for lymphedema	Arm measurements taken after bilateral breast surgery or a diagnosis of metastasis	1,121	2·00	RVC > 10% measured at least 3 months after surgery	184	0·08
Warren et al. [65]	USA	Women who underwent surgery for unilateral or bilateral breast cancer	Patients with preexisting lymphedema	1,476	2·12	RVC > 10% measured at least 3 months after surgery	202	0·06
Whelan et al. [62]	Canada	Women with invasive carcinoma of the breast and treated with breast-conserving surgery and SLNB or ALND and had positive axillary lymph nodes or negative axillary nodes with high-risk features	T4 tumors (clinical evidence of direct extension to chest wall or skin) or N2–3 nodes, distant metastasis, or serious nonmalignant disease that would preclude definitive radiation therapy	1,832	9·50	Not specified–clinical assessment	117	0·01
Youssef et al. [64]	England	Patients diagnosed with micrometastasis in the axilla	Neoadjuvant chemotherapy treatment	95	2·85	Not specified–clinical assessment	7	0·03
Darragh et al. [37]	Ireland	All patients undergoing a unilateral axillary procedure for breast cancer, preoperative measurements were required in addition to at least two postoperative readings	Previous bilateral procedures, previous axillary surgery, a pacemaker, a history of upper-limb DVT, arteriovenous fistulae, upper-limb fracture, and those who were pregnant	354	4·17	L-Dex values above the normal range or a change of more than 10 L-Dex units from baseline	23	0·02
Erdogan et al. [39]	Turkey	Surgery due to early-stage breast cancer	History of bilateral breast cancer, previous axillary surgery, history of lymphedema, pacemakers or metal implants, secondary conditions that may affect the fluid disruption (See Appendix)	37	1·00	L-Dex ratios greater than 10	5	0·14
Kaufman et al. [8]	USA	Breast cancer patients undergoing definitive breast cancer surgery with no limitation on the axillary management technique	Implantable electronic devices, pregnancy, renal failure, and heart failure	206	2·16	L-Dex ratios greater than 10 from baseline	0^a^	0^a^
Kilgore et al. [48]	USA	Breast cancer patients with unilateral disease undergoing treatments high-risk for BCRL	Not specified	146	1·69	BIS result of 2 standard deviations above baseline from preoperative assessment (> 10 points)	9	0·04
Laidley et al. [50]	USA	Some form of axillary staging [SLNB or ALND], preoperative L-Dex assessment, and ≥ two subsequent L-Dex assessments	Bilateral axillary surgery, previously diagnosis of BCRL, pregnancy, and implanted electronic cardiac device	326	1·81	A change in > 10 L-Dex units	11	0·02
Ridner et al. [17]	USA	Women ≥ 18 yoa with planned surgery for breast cancer, stage I–III invasive breast cancer or DCIS with at least one of these: mastectomy, axillary treatment, and taxane-based chemotherapy	Bilateral breast surgery, history of breast cancer; neoadjuvant chemotherapy; previous radiation to the breast, chest wall, or axilla; implanted medical device; previous lymphedema treatment; and other conditions (see Appendix)	259	1·52	An elevated L-Dex score of > 10 units from baseline	2	0·01
Whitworth et al. [63]	USA	Patients with breast cancer undergoing surgery (either breast conservation or mastectomy), ALND or SLMB	Bilateral disease, electronic devices (i·e·, pacemakers), pregnancy, renal failure, and heart failure	596	1·42	L-Dex score greater than 10 points from baseline	18	0·02
Armer et al. [15]	USA	Women over 18, with cT0-T4, NI-2, M0 breast cancer, fine needle aspiration or core needle biopsy of axillary node documenting nodal metastasis at diagnosis	Prior ipsilateral axillary surgery, or prior SLNB; patients who had bilateral breast cancer, current limb infection, lymphangitis, or any other condition that would affect testing	488	3·00	2-cm increase from a tape measure, compared to baseline and/or the contralateral arm	368	0·25
Ay et al. [22]	Turkey	Patients who underwent breast cancer surgery for stage II or III disease	Upper-limb trauma during the pre- and postoperative period, known vascular disease, serious thromboembolic event, neoadjuvant chemotherapy/ radiotherapy, and other conditions (See Appendix)	5,064	5·33	A difference of > 5% in circumference between the arms postoperatively	1008	0·04
Ballal et al. [24]	Netherlands	Patients having surgical procedures for malignant breast disease	Inadequate recorded information, previously undergone ipsilateral breast cancer surgery to the axilla, or undergoing tissue flap reconstructions	745	1·00	An increase in the ipsilateral arm compared to the contralateral arm of more than 2 cm at any one point	61	0·08
Benevento et al. [27]	Italy	Women over the age 25, absence of coagulopathy and/or liver disease, BMI = < 35, indication for ALND	Undergone previous breast surgeries and those who did not meet inclusion criteria	60	1·00	An increase in the ipsilateral arm compared to the contralateral arm of more than 2 cm at any one point	5	0·08
Blaney et al. [16]	Ireland	Women, aged 18–99 years, newly diagnosed with stages I–III unilateral breast cancer from specialist diagnostic breast clinics in Belfast City Hospital and Ulster Hospital	History of breast cancer or lymphoedema, prior severe trauma/surgery upper limb, pacemaker/ metal implant, no baseline assessment, Stage IV disease, and other additional conditions (see Appendix)	71	1·00	Tape measurement: a ≥ 5% increase in proximal, distal or total percentage volume difference (PVD) from baseline	28	0·39
Lorek et al. [51]	Poland	Patients with primary operative breast cancer who had received the SLNB procedure in combination with wide local excision (WLE) or simple mastectomy, or had SLNB prior to induction treatment	Not specified	303	2·13	A 10% difference between the limbs	9	0·01
McLaughlin et al. [52]	USA	Patients from Mayo Clinic's registry	Did not complete 12 month follow-up	120	1·00	10% increase in the ipsilateral arm when compared with the changes in the contralateral arm	12	0·10
Ochalek et al. [54]	Poland	Women from previously conducted RCT following breast cancer surgery and axillary nodes removed	Symptoms and/or signs of infection in affected limb, signs of heart or renal failure, preoperative LE, previous bilateral lymph node dissection, and other conditions (see Appendix)	45	1·00	A volume increase > 10% compared with the volume before surgery	7	0·16
Ridner et al. [17]	USA	Women ≥ 18 yoa with planned surgery for breast cancer, stage I–III invasive breast cancer or DCIS with at least one of these: mastectomy, axillary treatment, and taxane-based chemotherapy	Bilateral breast surgery, history of breast cancer; neoadjuvant chemotherapy; previous radiation to the breast, chest wall, or axilla; implanted medical device; previous lymphedema treatment; and other conditions (see Appendix)	239	1·47	volume change in the at-risk arm ≥ 10% above the presurgical baseline	10	0·03
Soran et al. [58]	USA	Patients who underwent ALND for BC in LE monitoring program	Patients with preoperatively diagnosed clinical or subclinical LE and prior trauma on the operated upper limb	180	1·76	Girth difference of ≥ 2·0 cm in the involved limb versus the uninvolved limb	22	0·07
Wetzig et al. [61]	Australia	Women with unifocal breast cancers < 3 cm diameter and clinically negative lymph nodes	Not specified	1,088	5·00	An increase of 15% or more from baseline in upper limb volume	28	0·01

^a^Study with 0 incidence was replaced with 0.5 for meta-analysis

For full descriptions, see Online appendix Table 2 was consolidated for space. *LE *lymphedema, *BCRL *Breast cancer-related lymphedema, *ALND* Axillary lymph node dissection, *BC* Breast cancer, *ILVD *Interlimb volume difference, *SLNB* Sentinel lymph node biopsy, *BIA *Bioimpedance assessment, *RVD *Relative volume change, *DVT *Deep vein thrombosis, *BIS *Bioimpedance spectroscopy, *PVD *Percentage volume difference, *WLE *Wide local excision

**Fig. 2:**
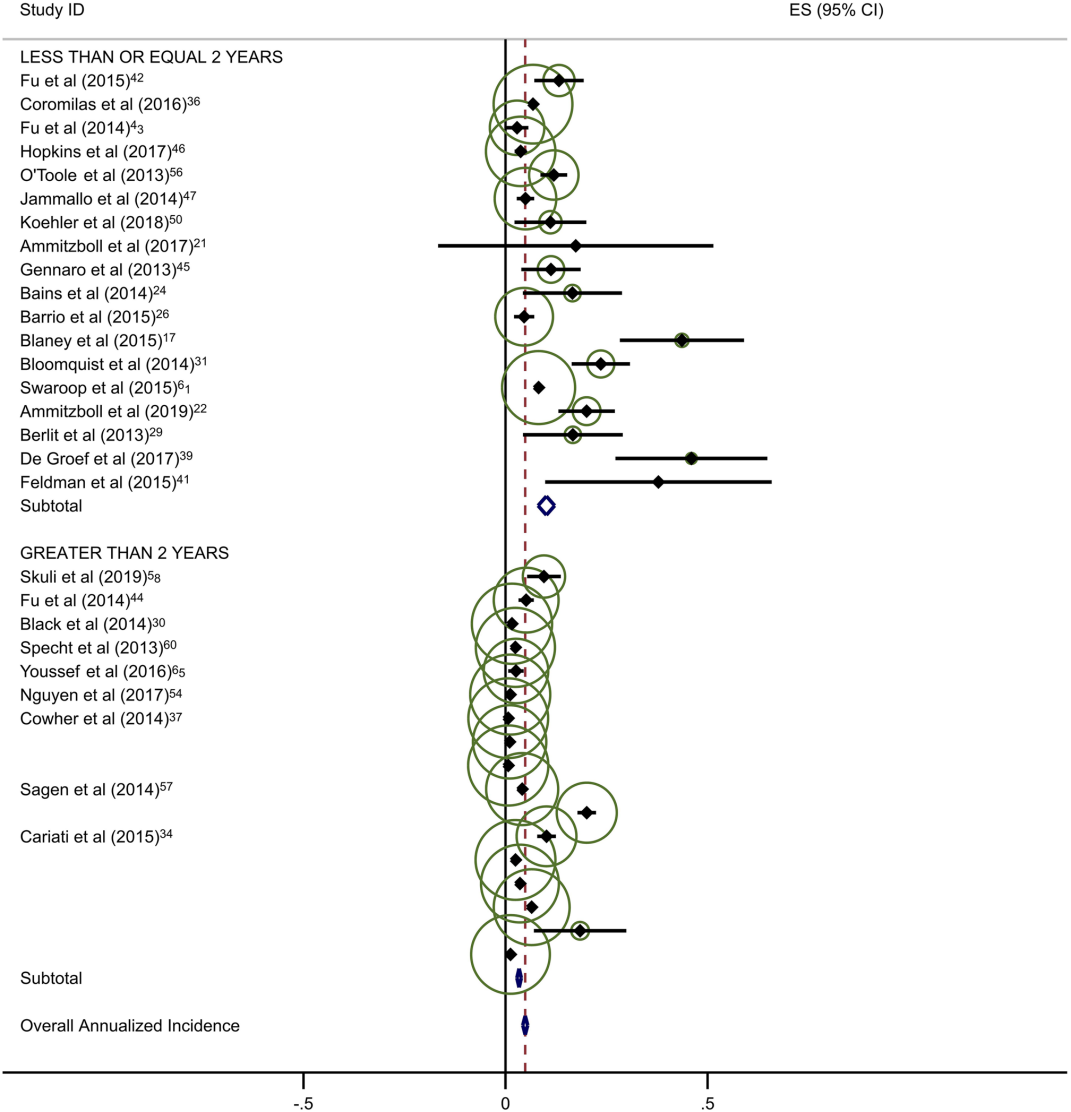

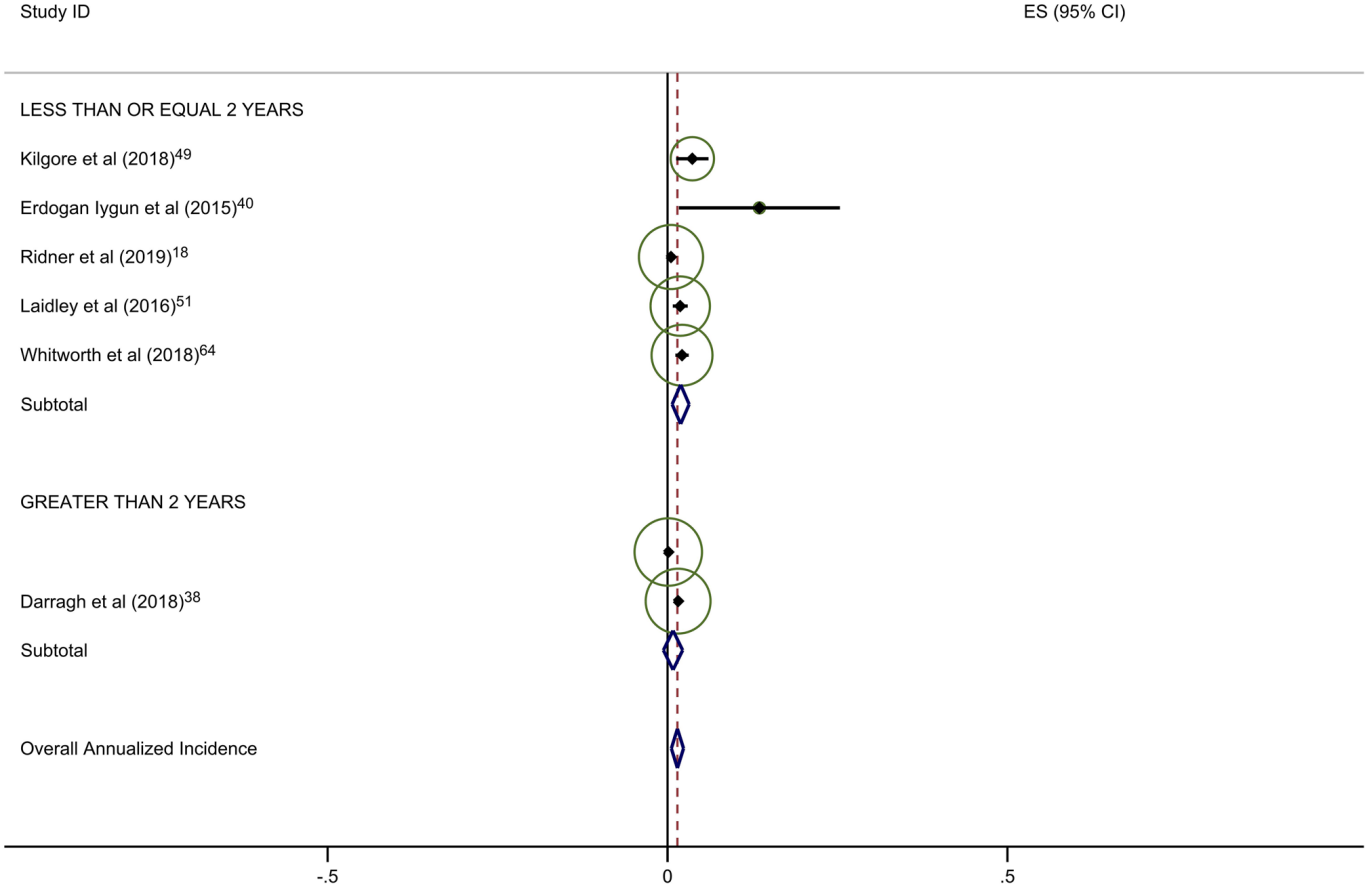

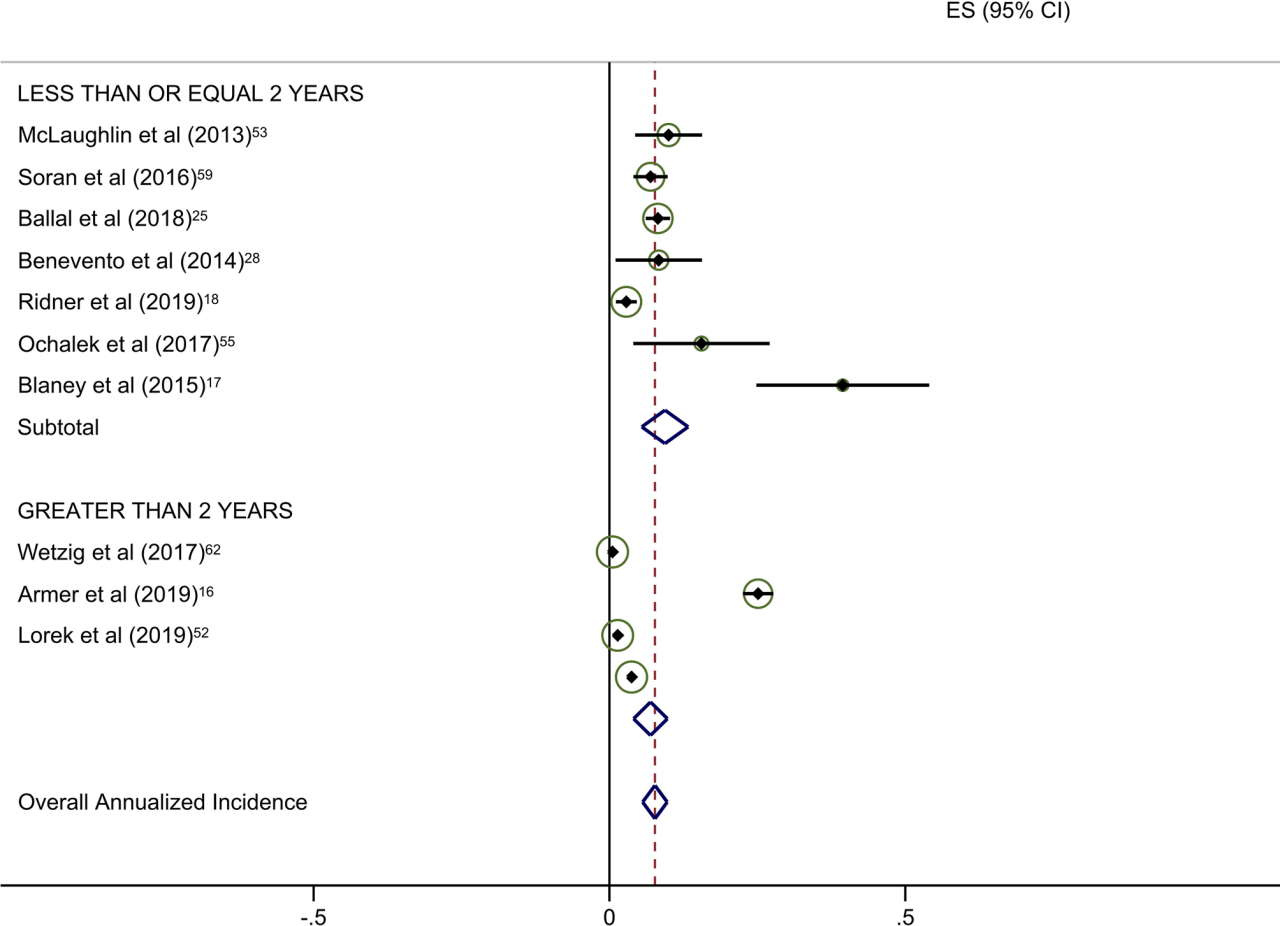
**a** Background studies with 2 year cut point. **b** BIS studies with 2 year cut point. **c** Circumference studies with 2 year cut point. Each study is represented by its estimated annualized incidence. The red dotted line is the overall pooled estimated annualized incidence. The light green circles represent the weight for each study to calculate the overall annualized incidence rate and are from the random-effects analysis. The diamond shapes are the variances for each cut point as well as the overall study variance

Estimates of average annual incidence varied significantly across studies, from 0·2% to 39·4%. Pooled summary estimates of annualized incidence were highest for RCTs and lowest for retrospective studies (Table 3). The pooled estimate for cumulative incidence of BCRL was 12.9% (95% CI: 11·3–14·4), 17.0% (95% CI: 10·3–23·7), and 3.1% (95% CI: 1·3–4·9) for background, circumference, and BIS, respectively. The annualized incidence was 4.9% (95% CI: 4·3–5·5), 7.7% (95% CI: 5·6–9·8), and 1.5% (95% CI: 0·6–2·4) for background, circumference, and BIS, respectively. Monitoring with BIS was associated with a significantly lower rate of progression to BCRL compared with rates in both background and circumference-monitored populations. Specifically, relative rates of progression in BIS-monitored patients were reduced by 69% compared to background rates and 81% compared to circumference-monitored populations based on overall pooled rates. This significant reduction in progression to BCRL among BIS-monitored patients was observed regardless of study duration or study type, with the exception of comparison to circumference-monitored patients in RCT studies. All differences in rates were statistically significant at *p* < 0·05, with a few exceptions as presented in Table 3 (Fig. 2a–c).

**Table 3:** Annualized incidence of breast cancer-related lymphedema, by study type

	No intervention (background rate)		Monitoring with BIS				Monitoring with circumference				Comparative rate ratio	
	Included studies (*n*)	Incidence (%; 95% CI)	Included studies (*n*)	Incidence (%; 95% CI)	Rate ratio^a^	*p*-value	Included studies (*n*)	Incidence (%; 95% CI)	Rate ratio^b^	*p*-value	BIS vs. circumference	*p*-value
Overall pooled estimate	35	4·9 (4·3–5·5)	7	1·5 (0·6–2·4)	0·31	< 0·001	11	7·7 (5·6–9·8)	1·57	< 0·001	0·19	< 0·001
By follow-up duration												
≤ 2 years	18	10·1 (8·0–12·2)	5	1·9 (0·7–3·2)	0·19	< 0·001	7	9·4 (5·5–13·3)	0·93	0·36	0·20	< 0·001
> 2 years	17	3·4 (2·8–3·9)	2	0·8 (− 0·6–2·2)	0·24	< 0·001	4	6·9 (4·1–9·8)	2·03	< 0·001	0·12	< 0·001
By study type												
Prospective	19	7·9 (6·1–9·8)	4	2·1 (0·0–4·1)	0·27	< 0·001	6	11·0 (5·8–16·3)	1·39	< 0·001	0·19	< 0·001
Randomized clinical trials	6	10·1 (6·1–14·0)	1	0·5 (− 0·2–1·2)	0·05	< 0·001	3	7·1 (0·4–13·8)	0·70	0·04	0·07	< 0·001
Retrospective	10	3·5 (2·7–4·4)	2	1·6 (1·1–2·2)	0·46	< 0·001	2	5·8 (1·5–10·2)	1·66	< 0·001	0·28	< 0·001

^a^BIS versus no intervention

^b^Circumference versus no intervention

*BIS* Bioimpedance spectroscopy

**Fig. 3:**
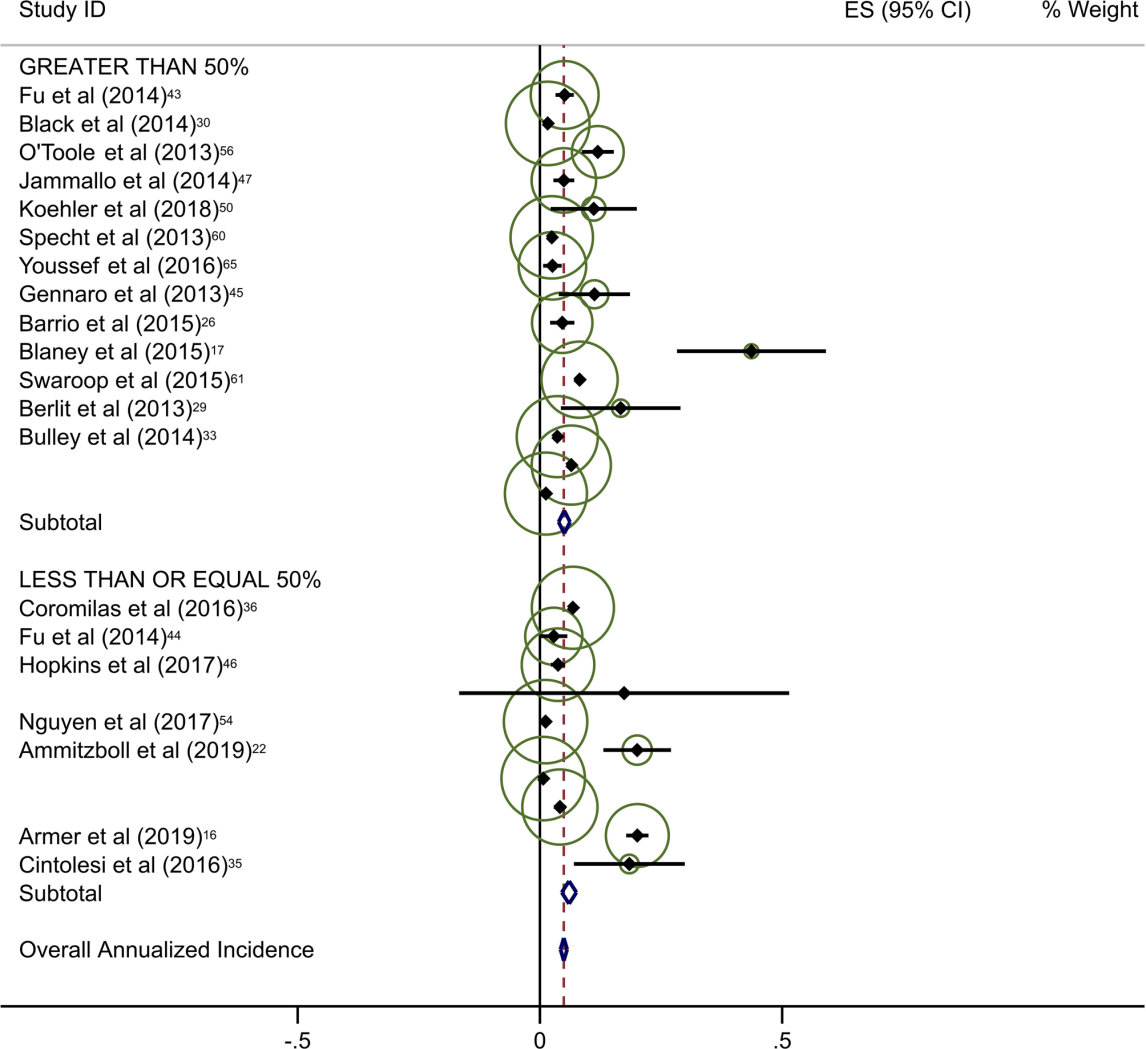

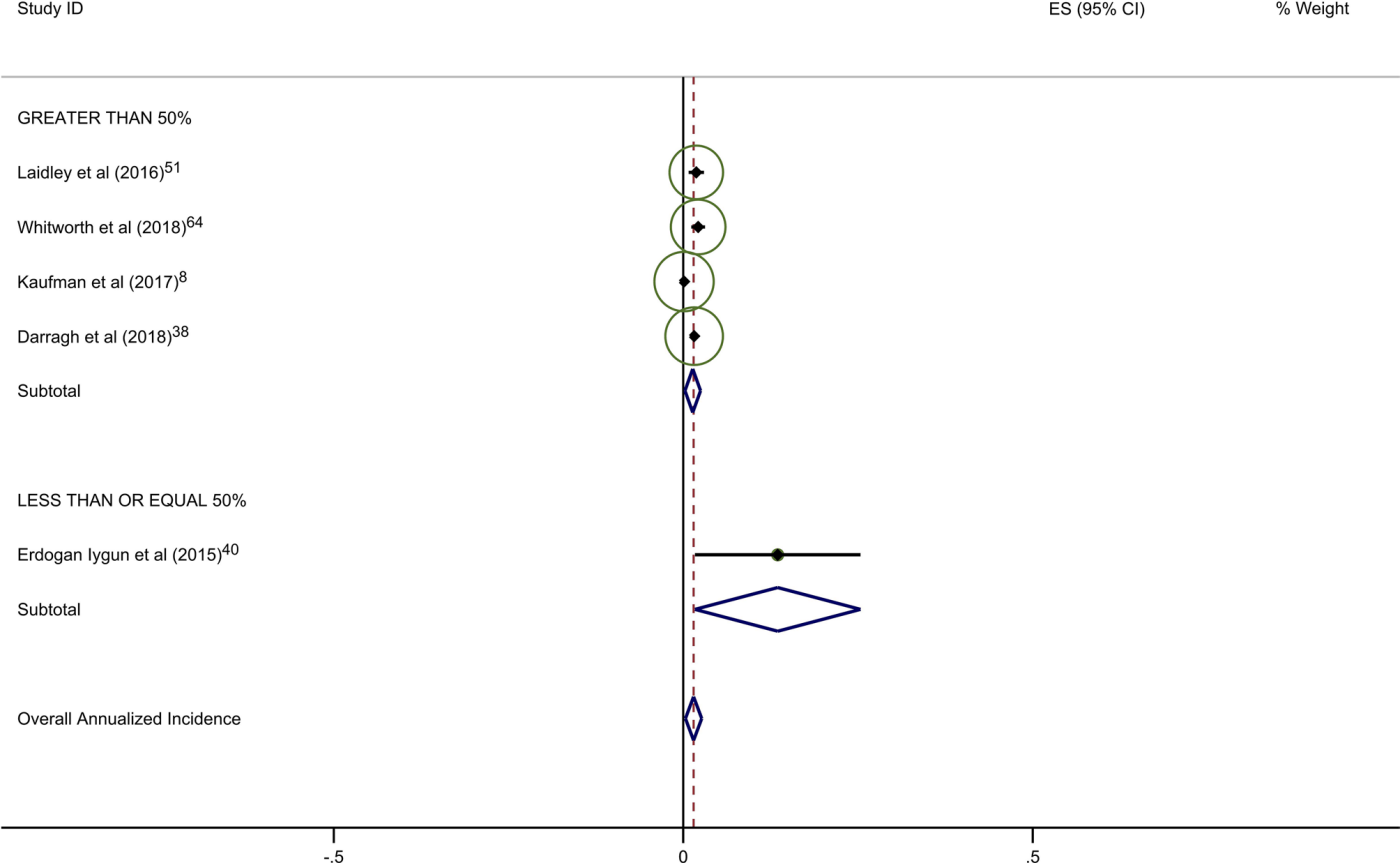

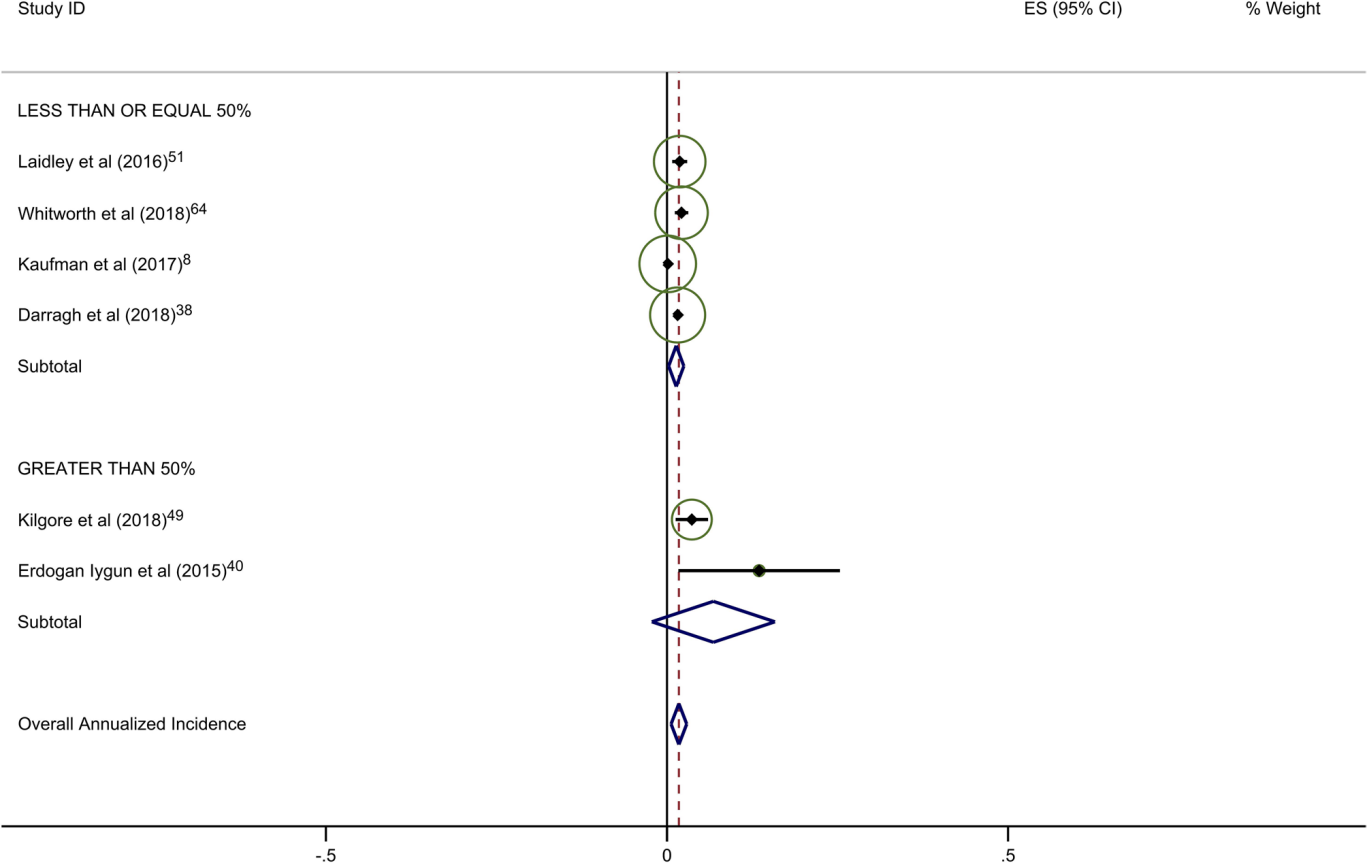

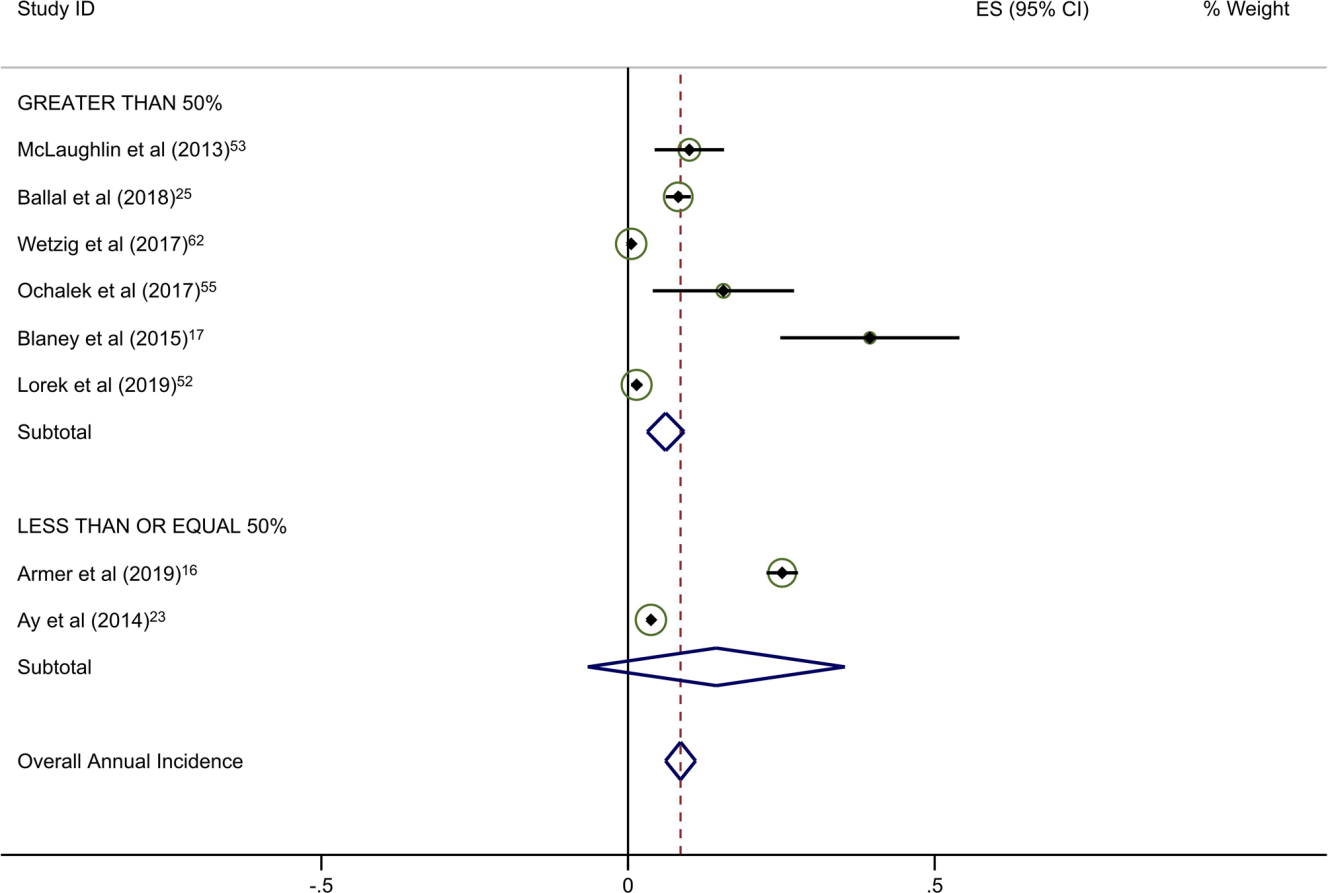

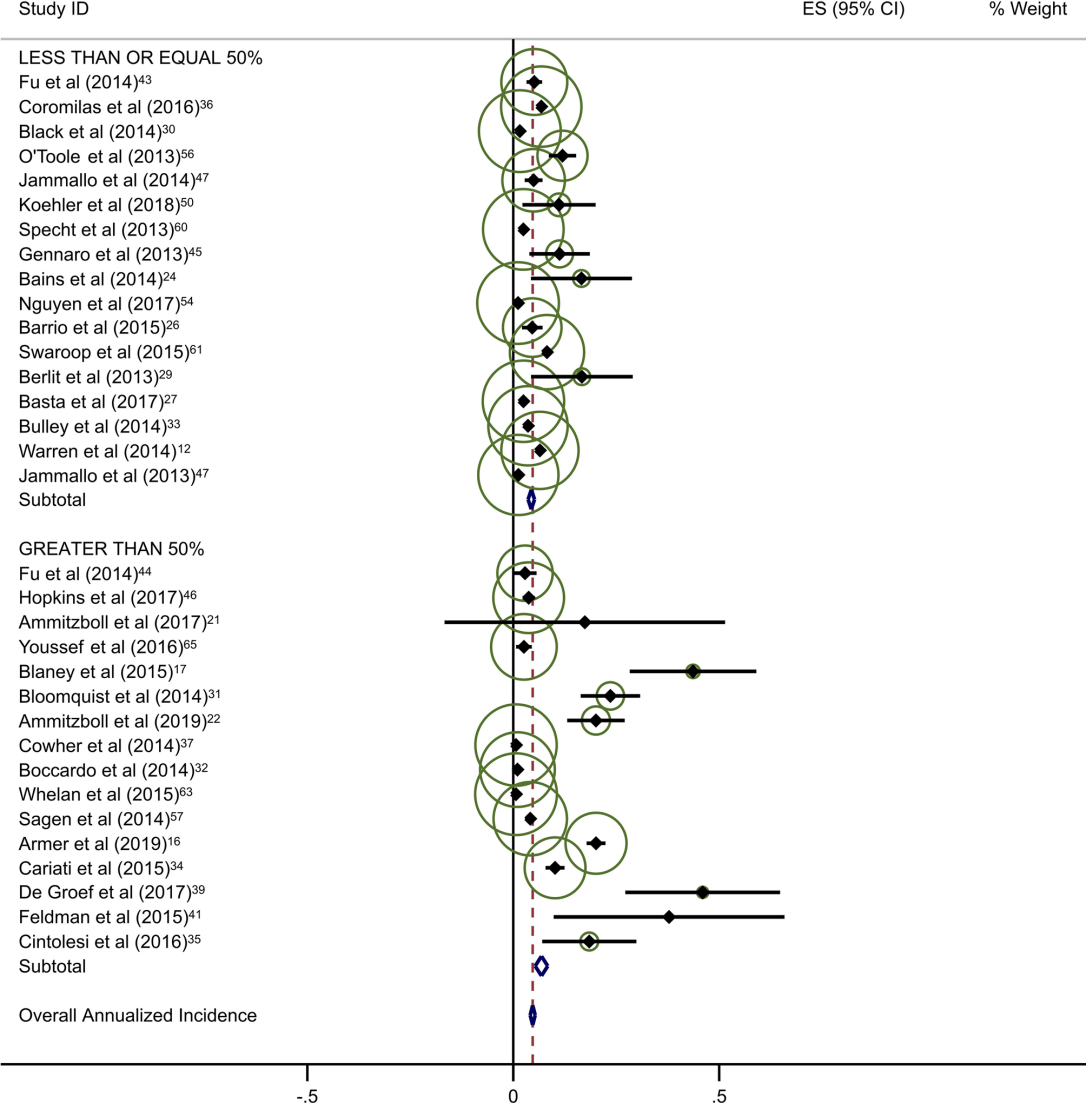

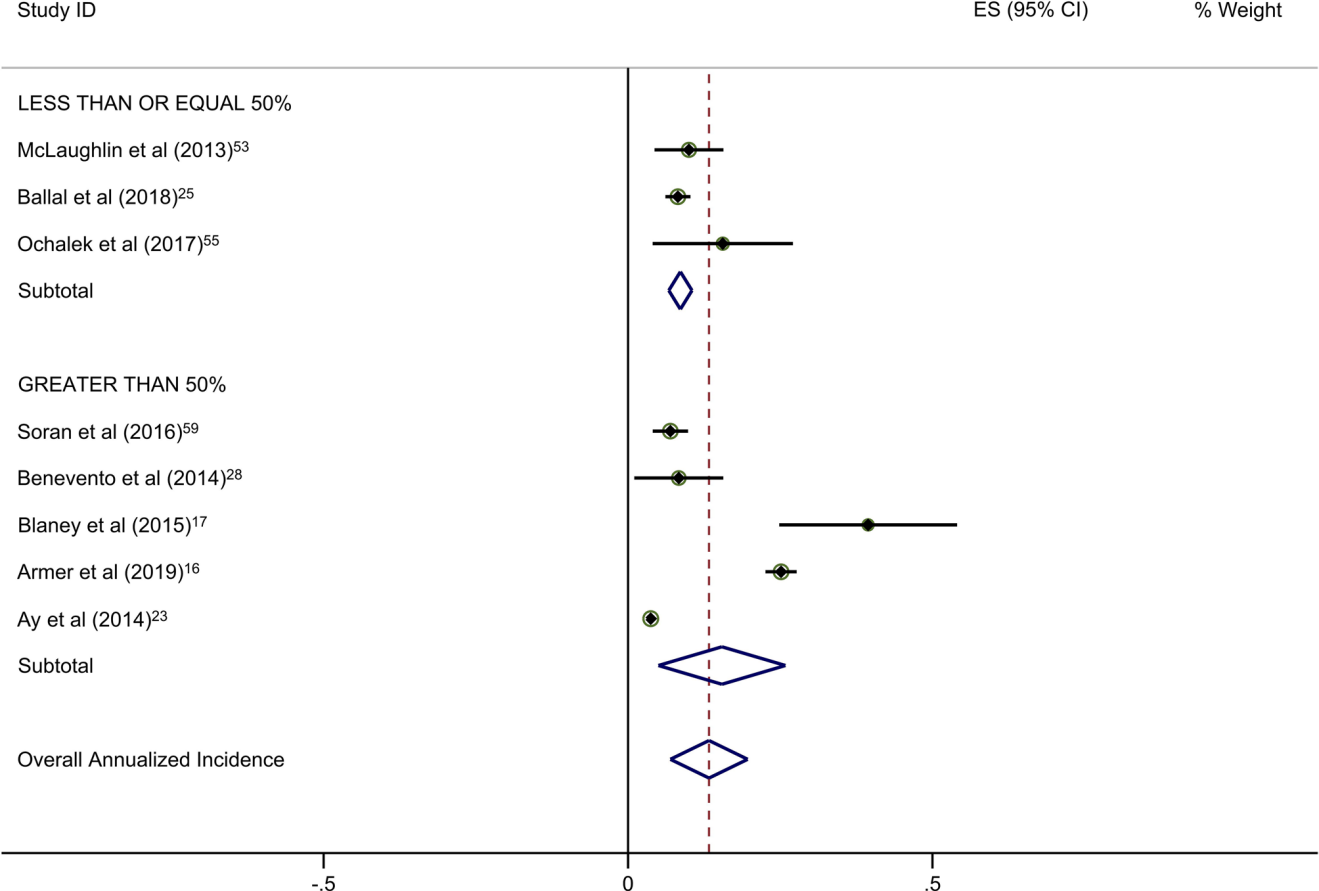
**a** Background studies combined for SLNB and ALND. **b** BIS studies combined for SLNB and ALND. **c** Circumference studies with SLNB and ALND. Each study is represented by its estimated annualized incidence. The red dotted line is the overall pooled estimated annualized incidence. The light green circles represent the weight for each study to calculate the overall annualized incidence rate and are from the random-effects analysis. The diamond shapes are the variances for each cut point as well as the overall study variance

Forty-five studies provided data on the percentage of patients receiving ALND. Overall rates of clinical BCRL were higher in studies with a majority (> 50%) of patients undergoing ALND (Table 4). This difference was observed for all three study arms, although it was most pronounced for BIS-monitored patients (Fig. 3a–c). BIS-monitored patients had a 56% reduced rate of progression compared to circumference-monitored patients in studies with > 50% ALND, and an 84% reduced rate of progression compared to circumference-monitored patients in studies with ≤ 50% ALND. BIS-monitored patients had a 68% reduced rate of progression compared to background studies in patients with ≤ 50% ALND. Forty-three studies provided data on the percentage of patients receiving mastectomy. Rates of clinical BCRL were lower in BIS and circumference-monitored studies with a high rate (> 40%) of patients receiving mastectomy compared to those with ≤ 40% of patients, but not for the background group. BIS-monitored patients had a 79% reduced rate of progression compared to circumference-monitored patients in studies with > 40% mastectomy, and a 7% reduced rate of progression compared to circumference-monitored patients in studies with a ≤ 40% mastectomy (not statistically significant) (Table 4). BIS-monitored patients had a 75% reduced rate of progression compared to background rates for patients with > 40% mastectomy.

**Table 4:** Subgroup Analyses

	No intervention (background rate)		Monitoring with BIS				Monitoring with circumference				Comparative rate ratio	
	Included studies (*n*)	Annualized Incidence (%; 95% CI)	Included studies (*n*)	Annualized Incidence (%; 95% CI)	Rate Ratio^a^	*p*-value	Included studies (*n*)	Annualized incidence (%; 95% CI)	Rate ratio^b^	*p*-value	BIS vs. circumference	*p*-value
Axillary lymph node dissection (ALND)												
≤ 50%	17	4·4 (3·6–5·2)	4	1·4 (0·2–2·5)	0·32	< 0·001	3	8·6 (6·7–10·5)	1·95	< 0·001	0·16	< 0·001
> 50%	16	6·8 (5·3–8·4)	2	6·8 (− 2·2–15·8)	1·00	1·0	5	15·4 (5·0–25·8)	2·26	< 0·001	0·44	< 0·001
Sentinel lymph node biopsy (SLNB)												
≤ 50%	10	6·0 (4·5–7·5)	1	13·5 (1·7–25·4)	2·25	0·06	2	14·4 (− 6·6–35·4)	2·40	< 0·001	0·94	0·88
> 50%	15	5·0 (3·8–6·2)	4	1·4 (0·2–2·5)	0·28	< 0·001	6	6·1 (3·1–9·1)	1·22	< 0·001	0·23	< 0·001
Mastectomy												
≤ 40%	17	4·1 (3·4–4·8)	1	13·5 (1·7–25·4)	3·29	0·004	4	14·5 (2·1–26·8)	3·54	< 0·001	0·93^¥^	0·87
> 40%	15	5·2 (3·9–6·6)	3	1·3 (− 0·2–2·8)	0·25	< 0·001	3	6·1 (2·7–9·5)	1·17	< 0·001	0·21	< 0·001

All rate ratios are statistically significant at *p* < 0·05 unless otherwise indicated

^a^BIS versus no intervention

^b^Circumference versus no intervention

*BIS* Bioimpedance spectroscopy

With respect to the potential for follow-up bias, the rates of clinical BCRL differed significantly for studies with average or median follow-up of ≤ 2 years versus those with > 2 years of follow-up (Table 3). The pooled rate of BCRL in studies with follow-up of ≤ 2 years was more than double that of longer studies for background and BIS studies (Fig. 2a, b) but the difference was not as pronounced in tape measurement studies (Fig. 2c).

## Discussion

The results of this meta-analysis demonstrate that patients followed with BIS surveillance were significantly less likely to develop chronic BCRL annually (and overall) as compared to the background rate and tape measurement. This difference was not only statistically significant but clinically as well; the annualized BCRL incidence rate was 69% (3.4% absolute difference) lower than the observed background rate and 81% (6.2% absolute difference) lower than that observed with tape measurement. Importantly, patients monitored with BIS had a lower annualized incidence even in high-risk populations (> 40% mastectomy and > 50% ALND). Similarly, a clinically significant difference in the cumulative BCRL incidence rate was noted with a 9.8% absolute reduction compared to observed background rate and a 13.9% absolute reduction as compared with tape measurement. While there were differences in rates of ALND between populations, when comparing studies with > 50% of patients undergoing ALND (Table 4), the benefits of BIS were confirmed (Annualized Incidence 6.8% vs. 15.4% with circumference, comparative ratio 0.44). It is important to recognize that screening techniques such as BIS do not directly reduce rates of BCRL; rather, these findings support that BIS is able to identify changes in fluid content earlier within the patient’s limb prior to the development of BCRL, and therefore, the benefit of BIS monitoring is to trigger early intervention in breast cancer patients at risk for chronic BCRL. This earlier diagnosis and intervention along with informing patients likely contribute to the reduction in BCRL allowing for the reversal of the increased fluid volume preventing chronic BCRL which is of substantial clinical importance. [12].

Our results are consistent with previous studies. This study builds on and supports the meta-analysis conducted by DiSipio et al. [4] The cumulative incidence observed in background rate studies, 12.9% (95% CI 11·3–14·4), is smaller but in a similar range to the cumulative incidence observed by DiSipio et al. (16·6%, 95% CI 13·6–20·2) and is likely consistent with changes in clinical practice over time; a higher rate of annual and cumulative BCRL was seen with circumference as compared to background and this may reflect that, consistent with DiSipio et al., all studies that used non-standard methods (ex. perometry, volume calculation) were included in the background group. Subgroup analyses were also consistent with prior research in identifying ALND as a risk factor for BCRL. Mastectomy as a risk factor was not confirmed in the background rate pooled estimates, although annualized incidence was higher in studies with more than 40% of patients undergoing mastectomy; this may reflect the increasing use of mastectomy with SLNB (and not ALND) as compared to previous studies. However, for both BIS and tape measurement monitoring studies, the annualized rate of BCRL was lower when the rate of mastectomy was higher (Table 4).

BIS monitoring enables early detection of changes in fluid in affected limbs, particularly in high-risk patient subgroups; additionally, despite potential concerns regarding challenges with implementation, studies have demonstrated the ability implementing this into a standard practice with clinical guidelines available as well [66, 67]. Early detection can be used to trigger interventions, typically consisting of some combination of compression garments, massage, and physical therapy, to prevent development of chronic BCRL. Such prevention measures improve patient quality of life and reduce the public health burden of chronic BCRL [1,^ 6^,^ 7^, 9]. Compared to no monitoring, BIS-monitored studies had an overall reduced chronic BCRL relative rate of 59%. Compared to tape measurement of circumference, BIS-monitored studies had a reduced relative rate of over 80%. These results provide further evidence in favor of BIS in preventing progression to BCRL in comparison to currently accepted methods of monitoring, possibly due to higher sensitivity of BIS to subclinical volume changes [5,^ 8^, 10]. There is now ample evidence that active monitoring of BCRL significantly reduces the risk of progression to BCRL. While this study compared the effectiveness of monitoring with BIS versus tape measurement for the development of BCRL, future studies should compare BIS to other accepted surveillance methods individually in a prospective study design evaluating high-risk patients. Additionally, recent data and the PREVENT trial have reduced the change initiating a trigger for intervention from 10 to 6.5, potentially further increasing the sensitivity of BIS to detect subclinical BCRL [17, 68].

A key strength of this study is the robust statistical analyses performed. Multiple risk factors of interest were examined for increased risk of BCRL. However, several important limitations must be noted. First, there were relatively few studies (*n* = 7) that used BIS to monitor progression to chronic BCRL. Second, only 28% of BIS patients had ALND, versus 73% of circumference patients, suggesting the latter group was at disproportionately greater risk of BCRL. Third, data on patient BMI in many studies were either not available or were not presented continuously. Fourth, most studies did not provide data specifically on taxane-based chemotherapy or RNI (radiation fields including axilla vs axilla + supraclavicular vs. supraclavicular alone), which are also risk factors for BCRL. It would have been advantageous to include subgroup analyses on BMI, taxane-base chemotherapy, and RNI, as they are known to increase patient’s risk for BCRL. Definitions of BCRL varied across studies. However, there are no standard clinical cut-points for many of these measures other than BIS [17]. For the BIS studies, in particular, there was greater consistency, with BCRL defined as an increase of 10 units or more in L-Dex scores. Finally, the background group was heterogeneous, though consistent with the previous meta-analysis [4]. A sensitivity analysis was conducted on the background studies to evaluate the BCRL rates for studies with no active monitoring in comparison to other commonly performed monitoring methods (e.g., water displacement) that were included in the background. With no monitoring (*n* = 11), the annualized incidence rate for BCRL was 3·7%. Background studies with other methods of monitoring (*n* = 24) had an annualized incidence rate of 7·6%. This difference indicates the possibility of detection bias when evaluating the low incidence rate of the patients in the no monitoring group. Without active monitoring, it is highly possible for patients to develop BCRL and go undiagnosed, which can bias the results.

## Electronic supplementary material

Below is the link to the electronic supplementary material.

Supplementary file1 (docx 29 kb)
