# Supplementary material for: The impact of monitoring techniques on progression to chronic breast cancer-related lymphedema: a meta-analysis comparing bioimpedance spectroscopy versus circumferential measurements
Source: Breast Cancer Res Treat. 2020 Nov 27;185(3):709–40. doi: 10.1007/s10549-020-05988-6 (PMC7921068; doi:10.1007/s10549-020-05988-6)
Supplement: Supplementary file 1 — Supplementary file1 (docx 29 kb) [file 10549_2020_5988_MOESM1_ESM.docx]

**Appendix A: Full Description of Study Characteristics**

| **Study** | **Location** | **Inclusion Criteria** | **Exclusion Criteria** | **Total**  **Patients (n)** | **Duration of Follow-Up**  **(Mean/Median), Years** | **LE Definition** | **Patients Progressed to BCRL (n)** | **Annual Rate of Progression**  **(Patients progressed/ person-time)** |
| --- | --- | --- | --- | --- | --- | --- | --- | --- |
| Ammitzboll et al (2017) | Denmark | Women aged 18-75, operated with ALND for unilateral primary BC, able to transport themselves to the hospital, and physically and mentally able to participate in the exercise intervention | Previous ALND either side, primary breast reconstruction, metastatic disease, and history of lymphoedema | 6 | 0.96 | A change in interlimb volume difference (ILVD), in which the affected arm increased  more than 3% relative to the other arm, if accompanied by an increased score on the numeric rating scale (NRS) for cardinal symptoms of swelling, and if two of four clinical criteria were fulfilled compared to unaffected arm: (1) increased thickness of the skin; (2) decreased contours in the area of the medial epicondyle, wrist and hand; (3) decreased visibility of veins; and (4) pitting oedema | 1 | 0.17 |
| Ammitzboll et al (2019) | Denmark | Women age 18 to 75 years, primary unilateral BC, ALND, no distant metastases, no previous ALND on contralateral side, no previous history of arm lymphedema, and ability to participate in group exercise regimen | Not specified | 158 | 1 | >3% increase in ILVD | 32 | 0.20 |
| Armer et al (2018) | USA | Women over 18 years of age, with cT0-T4, NI-2, M0 breast cancer, fine needle aspiration or core needle biopsy of axillary node documenting nodal metastasis at diagnosis, prior to neoadjuvant chemotherapy | Prior ipsilateral axillary surgery, or prior SLN surgery/excisional lymph node biopsy for pathologic confirmation of axillary status; patients who had bilateral breast cancer, current limb infection, lymphangitis, or any other condition that would affect testing. | 488 | 3 | A volume increase >10% compared to baseline and /or the contralateral arm | 294 | 0.20 |
| Bains et al (2014) | United Kingdom | Invasive breast cancer and undergoing axillary lymph node surgery | Not specified | 38 | 1.12 | 5-10% volume increase from Perometer readings and clinical signs of BCRL | 7 | 0.17 |
| Barrio et al (2015) | USA | Female patients age >18, newly diagnosed invasive or in situ breast carcinoma, and planned unilateral axillary surgery with either SLNB or ALND | Not specified | 186 | 1.52 | Relative arm volume difference (RAVD) of >10% | 13 | 0.05 |
| Basta et al (2017) | USA | Patients with breast cancer diagnosis and mastectomy | Patients not undergoing mastectomy or undergoing mastectomy outside the specified study time frame, those with documented lymphedema before mastectomy, and those with less than 12 months of follow-up or mortality within 12 months of mastectomy | 3136 | 4.2 | ICD-9 DX codes 457.0 and 457.1 | 325 | 0.02 |
| Berlit et al (2013) | Germany | Used study sample from previously conducted study | Not specified | 42 | 1 | N/A – used circumference measurements and defined in previous study | 7 | 0.166667 |
| Black et al (2014) | USA | Pathologically node-negative disease and a documented axillary surgical procedure on women aged 66 years or older | Not specified | 31274 | 5 | ICD-9 DX codes 457.0 and 457.1 for Lymphedema | 2501.533 | 0.07999 |
| Blaney et al (2014) | Ireland | Women, aged 18–99 years, newly diagnosed with stages I–III unilateral breast cancer from specialist diagnostic breast clinics in Belfast City Hospital and Ulster Hospital | Previous history of breast cancer, prior severe trauma/surgery upper limb, patients fitted with cardiac device/metal implant, unable to obtain baseline assessment - referral received after/on day of surgery, patients with stage IV disease/locally advanced, Dementia/Alzheimer's/ memory/communication difficulties, patients with renal failure/heart failure, not appropriate for surgery, bilateral breast cancer, patients reporting pre-operative swelling of the upper limb, previous history of lymphoedema, did not attend baseline assessment | 71 | 1 | BIA classification: L-Dex score of >10 or a 10 U increase from baseline | 31 | 0.44 |
| Bloomquist et al (2014) | Denmark | Breast cancer patients who had participated in the exercise intervention, had received at least one cycle of chemotherapy for advanced disease or as adjuvant treatment, had a WHO performance status of 0 or 1 and otherwise had been approved to participate by the treating oncologist | Patients that had recurrent cancer, deceased, or diagnosed BCRL prior to participation in "Body & Cancer" | 149 | 1.17 | An interlimb difference of >2 cm at to two or more measures | 41 | 0.24 |
| Boccardo et al (2013) | Italy | Candidates for Lymphatic Microsurgical Preventing Healing Approach (LYMPHA) procedure during ALND | No afferent lymphatics could be found and because of massive meta- static disease | 74 | 4 | Missing – not specified. Volumetry used | 3 | 0.01 |
| Bulley et al (2014) | United Kingdom | Women who had completed treatment (surgery, chemotherapy and radiotherapy), and who were attending review appointments | If they had recurrence, or were unable to complete non-translated questionnaires | 613 | 5.25 | Limb volume difference (LVD) was 10% or greater (unilateral treatment only) | 115 | 0.04 |
| Cariati et al (2015) | England | All patients diagnosed with breast cancer who underwent ALND | Not specified | 273 | 2.67 | >10 % arm volume increase compared to contralateral arm by perometer or subjective assessment | 74 | 0.10 |
| Cintolesi et al (2016) | United Kingdom | Women newly diagnosed with breast cancer | Not specified | 26 | 2.08 | Clinical Assessment for any of the following: (i) decreased visibility of subcutaneous veins on the ventral forearm and dorsal hand; (ii) smoothing or fullness of the medial elbow and distal upper arm contours; (iii) increased skin and subcutis thickness if the tissues are pinched between finger and thumb; and (iv) pitting edema upon application of thumb pressure for 60 seconds. In addition, the thickness of the posterior axillary fold (PAF) was assessed by pinch test. The volume of each upper limb changes from baseline by Perometer | 10 | 0.18 |
| Coromilas et al (2016) | USA | From SEER-Medicare database, women aged 65-90 with DCIS who underwent excisional breast procedure (BCS), simple mastectomy, total mastectomy with ALND, or skin-sparing mastectomy | Excluded patients with any code for invasive breast cancer | 10504 | 1 | ICD-9 DX codes 457.0 and 457.1 | 714 | 0.07 |
| Cowher et al (2014) | USA | Women undergoing mastectomy with CARE performed by the same surgeon | Not specified | 587 | 5.1 | Occupational therapist or surgeon’s note documenting a decreased function and/or quality of life due to arm swelling or pain | 20 | 0.01 |
| De Groef et al (2017) | Belgium | Women treated for a primary breast cancer, radiation therapy was terminated at least 3 months ago, pain at the upper limb region reaching a score of at least 40 of 100 on VAS during past week, and presence of myofascial dysfunctions at upper limb region | If they were not able to visit the hospital for therapeutic sessions and measurements for the entire duration, presence of shoulder pathologies for which surgical indications exist, or presence of current episodes of cancer or metastasis | 50 | 1 | >5.0% increase of relative arm volume difference compared to pre-surgical value | 23 | 0.46 |
| Feldman et al (2015) | USA | Female patients with breast cancer and documented axillary nodal metastasis undergoing planned axillary node dissection or modified radical mastectomy | Those not undergoing complete axillary node dissection, allergy to Lymphazurin blue dye, and pregnancy | 37 | 0.5 | More than 2 cm discrepancy in circumferential size measurements between the affected and unaffected arms or a change from baseline | 7 | 0.378378 |
| Fu et al (2014) | USA | Women who were over age 21, first time diagnosis of breast cancer (Stage I- III), and scheduled for surgical treatment, including lumpectomy or mastectomy, sentinel lymph node biopsy (SLNB) or axillary lymph node dissection ALND | Women with metastatic cancer (Stage IV), prior history of breast cancer and lymphedema, and bilateral breast cancer | 140 | 1 | 10 % LV increase from baseline in the ipsilateral arm compared with changes in the contralateral arm by perometer | 4 | 0.03 |
| Fu et al (2014) | USA | Patients with non-metastatic invasive breast cancer who had undergone mastectomy and either SLNB or ALND as their first nodal surgery | Under 18, lumpectomy, no nodal surgery, pN stage: N0, neo-adjuvant chemotherapy, cancer within 5 years | 151 | 3.63 | Not specified | 28 | 0.05 |
| Fu et al (2015) | USA | Women over the age of 21, first time diagnosis of breast cancer (stages 1-3), scheduled for surgical treatment including lumpectomy or mastectomy, sentinel lymph node biopsy or axillary lymph node dissection | Diagnosed with metastatic cancer, prior history of breast cancer and lymphedema, bilateral breast cancer, artificial knee or hip, kidney failure, heart failure | 136 | 1 | L-Dex ratios greater than 7.1. [BIA measurements] | 18 | 0.13 |
| Gennaro et al (2013) | Italy | Consecutive patients undergoing ARM with a diagnosis of axillary nodal involvement based on preoperative needle biopsy or a positive sentinel lymph node biopsy and scheduled for ALND | Not specified | 60 | 1.33 | Arm circumference measurements increasing by >2 cm, and/or based on subjective symptoms and/or comparative lymphoscintigraphy | 9 | 0.11 |
| Hopkins et al (2017) | USA | 321 of the CALGB 49907 patients were also enrolled in a QOL companion trial | Not specified | 321 | 2 | Physician reported BCRL; Progression not specified | 24 | 0.04 |
| Jammallo et al (2013) | USA | Patients undergoing treatment for primary breast underwent prospective lymphedema screening, had preoperative Perometer measurements and subsequent post-operative measurements corresponding with oncology follow-up visits | Patients who underwent bilateral breast surgery and measurements occurring after a patient was diagnosed with metastatic disease were also excluded | 787 | 2.25 | RVC >10% occurring >3 months post-operative | 22 | 0.01 |
| Jammallo et al (2014) | USA | Women undergoing treatment for newly diagnosed breast | Those who underwent bilateral breast surgery and those with metastatic disease were excluded from the analysis | 324 | 1.25 | RVC greater than or equal to 10% | 20 | 0.05 |
| Koehler et al (2018) | USA | Women having undergone early stage surgical breast cancer treatment (lumpectomy or mastectomy) with a minimum removal of one axillary lymph node by sentinel node biopsy | Previous surgical treatment for breast cancer or synchronous bilateral breast cancer, or a previous history of shoulder surgery, shoulder dysfunction, or history of upper extremity deep vein thrombosis | 36 | 1.5 | More than 10% difference in volume, >200 mL difference in volume, >2cm difference at any site on the upper extremity using circumferential measurements or an L-Dex value over 10. | 6 | 0.11 |
| Nguyen et al (2017) | USA | All incident breast cancer cases diagnosed in Olmsted County, MN residents in the Olmsted County Rochester Epidemiol- ogy Project Breast Cancer Cohort | +Not specified | 1794 | 10 | All cases with definite or probable lymphedema in the clinical notes | 209 | 0.01 |
| O’Toole et al (2013) | USA | Women diagnosed with primary breast cancer, underwent unilateral breast surgery, underwent pre-operative assessment, post-operative assessment, and at least one follow-up assessment >3 months after surgery | Patients with bilateral breast or axillary surgery, distant metastasis or recurrence | 308 | 1.36 | An arm volume increase of >5 % RVC, | 50 | 0.12 |
| Sagen et al (2014) | Norway | ages 35 to 75 years, diagnosed with early-stage primary breast cancer, surgery with breast ablation or breast conserving surgery with ALND or SLNB only | Too frail, inability to cognitively comprehend, metastasized breast cancer or other cancer, preoperative injuries, pathologic problems affecting upper limb function while performing tests | 391 | 2.5 | 10 % arm volume increase compared to contralateral arm by Perometer | 40 | 0.04 |
| Skuli et al (2018) | USA | A cohort of patients who participated in a 60- to 90-min SV after referral by patients’ medical oncology providers and occurred about 1-3 months after completion of locoregional therapy and initial systemic therapy | Not specified | 87 | 2.42 | N/A – retrospective chart review | 20 | 0.10 |
| Specht et al (2013) | USA | Women diagnosed with breast cancer | Patients who underwent bilateral breast or axillary surgery were excluded. | 1173 | 4 | RVC >10 % measured at least 3 months after surgery | 114 | 0.02 |
| Swaroop et al (2015) | USA | Women diagnosed with unilateral breast cancer who underwent surgery and prospective screening for lymphedema | Arm measurements taken after bilateral breast surgery or a diagnosis of metastasis | 1121 | 2 | RVC >10 % measured at least 3 months after surgery | 184 | 0.08 |
| Warren et al (2014) | USA | Women who underwent surgery for unilateral or bilateral breast cancer | Patients with pre-existing lymphedema | 1476 | 2.12 | RVC >10 % measured at least 3 months after surgery | 202 | 0.06 |
| Whelan et al (2015) | Canada | Women with invasive carcinoma of the breast who were treated with breast-conserving surgery and sentinel-lymph-node biopsy or axillary-node dissection and who had positive axillary lymph nodes or negative axillary nodes with high-risk features | T4 tumors (clinical evidence of direct extension to chest wall or skin) or N2–3 nodes (involvement of axillary nodes that are fixed or of internal mammary nodes), distant metastasis, or serious nonmalignant disease (e.g., cardiovascular or pulmonary) that would preclude definitive radiation therapy | 1832 | 9.5 | Not specified – clinical assessment | 117 | 0.01 |
| Youssef et al (2016) | England | Patients diagnosed with micrometatsis in the axilla | Neoadjuvant chemotherapy treatment | 95 | 2.85 | Not specified – clinical assessment | 7 | 0.03 |
| Darragh et al (2018) | Ireland | All patients undergoing a unilateral axillary procedure for breast cancer, pre-operative measurements were required in addition to at least two postoperative readings. | Patients who had bilateral procedures, previous axillary surgery, a pacemaker, a history of upper-limb DVT, arteriovenous fistulae, upper- limb fracture, and those who were pregnant | 354 | 4.17 | L-Dex values above the normal range or a change of more than 10 LDex units from baseline. | 23 | 0.02 |
| Erdogan et al (2015) | Turkey | Surgery due to early stage breast cancer | History of bilateral breast cancer, previous axillary surgery, history of lymphedema, secondary conditions such as pregnancy or cardiac or renal dysfunction that may affect the fluid disruption in the body, pacemakers or metal implants | 37 | 1 | L-Dex ratios greater than 10 | 5 | 0.14 |
| Kaufman et al (2017) | USA | Patients with breast cancer undergoing definitive breast cancer surgery (breast conservation or mastectomy) with no limitation on the axillary management technique | Implantable electronic devices (i.e., pacemakers), pregnancy, renal failure, and heart failure. | 206 | 2.16 | L-Dex ratios greater than 10 from baseline | 0* | 0* |
| Kilgore et al (2018) | USA | Breast cancer patients with unilateral disease undergoing treatments high-risk for BCRL | Not specified | 146 | 1.69 | BIS result of 2 standard deviations above baseline from preoperative assessment (>10 points) | 9 | 0.04 |
| Laidley et al (2016) | USA | Some form of axillary staging [sentinel lymph node biopsy (SLNB) or axillary lymph node dissection (ALND)], preoperative L-Dex assessment, and a minimum of two subsequent L-Dex assessments | Bilateral axillary surgery, previously documented diagnosis of BCRL, pregnancy, and implanted electronic cardiac device, such as a pacemaker | 326 | 1.81 | A change in >10 L-Dex units | 11 | 0.02 |
| Ridner et al (2019) | USA | Women ≥18 years of age with histologically confirmed breast cancer (invasive or ductal carcinoma in situ [DCIS]) with planned surgery, stage I–III invasive breast cancer or DCIS with at least one of the following: mastectomy, axillary treatment (ALND, SLNB with greater than 6 nodes, axillary radiation), and taxane-based chemotherapy | Bilateral breast surgery, a prior history of breast cancer; neoadjuvant chemotherapy; previous radiation to the breast, chest wall, or axilla; implanted medical device; conditions known to cause swelling (excluding pregnancy, congestive heart failure, chronic/acute renal disease, cor pulmonale, nephrotic syndrome, nephrosis, liver failure or cirrhosis, pulmonary edema, and thrombophlebitis or deep vein thrombosis in the arms); previous lymphedema treatment in either arm; uncontrolled intercurrent illness; psychiatric illness that would limit compliance with the study; and known allergy to electrode adhesives or compression fabrics | 259 | 1.52 | An elevated L-Dex score of > 10 units from baseline | 2 | 0.01 |
| Whitworth et al (2018) | USA | Patients with breast cancer undergoing surgery (either breast conservation or mastectomy), ALND or SLMB | Bilateral disease, electronic devices (i.e., pacemakers), pregnancy, renal failure, and heart failure. | 596 | 1.42 | L-Dex score greater than 10 points from baseline | 18 | 0.02 |
| Armer et al (2018) | USA | Women over 18 years of age, with cT0-T4, NI-2, M0 breast cancer, fine needle aspiration or core needle biopsy of axillary node documenting nodal metastasis at diagnosis, prior to neoadjuvant chemotherapy | Prior ipsilateral axillary surgery, or prior SLN surgery/excisional lymph node biopsy for pathologic confirmation of axillary status; patients who had bilateral breast cancer, current limb infection, lymphangitis, or any other condition that would affect testing. | 488 | 3 | 2-cm increase from a tape measure, compared to baseline and /or the contralateral arm | 368 | 0.25 |
| Ay et al (2014) | Turkey | Patients who underwent breast cancer surgery for stage II or III disease | Patients who had upper limb trauma during the pre- and postoperative period, known vascular disease, serious thromboembolic event, undergone neoadjuvant chemotherapy or radiotherapy, uncontrolled diabetes mellitus, cardiovascular disease that caused sufficient perfusion deficiency to delay recovery or a history of serious infection or surgery | 5064 | 5.33 | A difference of >5% in circumference between the arms postoperatively | 1008 | 0.04 |
| Ballal et al (2017) | Netherlands | Patients having surgical procedures for malignant breast disease | Inadequate recorded information, previously undergone ipsilateral breast cancer surgery to the axilla, or undergoing tissue flap reconstructions | 745 | 1 | An increase in the ipsilateral arm compared to the contralateral arm of more than 2 cm at any one point | 61 | 0.08 |
| Benevento et al (2014) | Italy | Women over the age 25, absence of coagulopathy and/or liver disease, BMI=<35, indication for ALND | Undergone previous breast surgeries and those who did not meet inclusion criteria | 60 | 1 | An increase in the ipsilateral arm compared to the contralateral arm of more than 2 cm at any one point | 5 | 0.08 |
| Blaney et al (2014) | Ireland | Women, aged 18–99 years, newly diagnosed with stages I–III unilateral breast cancer from specialist diagnostic breast clinics in Belfast City Hospital and Ulster Hospital | Previous history of breast cancer, prior severe trauma/surgery upper limb, patients fitted with cardiac device/metal implant, unable to obtain baseline assessment - referral received after/on day of surgery, patients with stage IV disease/locally advanced, Dementia/Alzheimer's/ memory/communication difficulties, patients with renal failure/heart failure, not appropriate for surgery, bilateral breast cancer, patients reporting pre-operative swelling of the upper limb, previous history of lymphoedema, did not attend baseline assessment | 71 | 1 | Tape measurement: a ≥5 % increase in proximal, distal or total percentage volume difference (PVD) from baseline | 28 | 0.39 |
| Lorek et al (2019) | Poland | Patients with primary operative breast cancer who had received the SLNB procedure in combination with wide local excision (WLE) or simple mastectomy, or had autonomous SLNB prior to induction treatment based on the SentiMag® method | Not specified | 303 | 2.13 | A 10% difference between the limbs | 9 | 0.01 |
| McLaughlin et al (2013) | USA | Patients from Mayo Clinic's registry | Did not complete 12 month follow-up | 120 | 1 | 10% increase in the ipsilateral arm when compared with the changes in the contralateral arm | 12 | 0.1 |
| Ochalek et al (2017) | Poland | Women from previously conducted RCT following breast cancer surgery and axillary nodes removed | Symptoms and/or signs of infection in the affected limb, signs of heart or renal failure, vein thrombosis, severe pulmonary insufficiency or liver disease, any evidence of active cancer or preoperative LE (≥10% difference in limb volumes), and history of bilateral lymph node dissection. | 45 | 1 | A volume increase >10% compared with the volume before surgery | 7 | 0.16 |
| Ridner et al (2019) | USA | Women ≥18 years of age with histologically confirmed breast cancer (invasive or ductal carcinoma in situ [DCIS]) with planned surgery, stage I–III invasive breast cancer or DCIS with at least one of the following: mastectomy, axillary treatment (ALND, SLNB with greater than 6 nodes, axillary radiation), and taxane-based chemotherapy | Bilateral breast surgery, a prior history of breast cancer; neoadjuvant chemotherapy; previous radiation to the breast, chest wall, or axilla; implanted medical device; conditions known to cause swelling (excluding pregnancy, congestive heart failure, chronic/acute renal disease, cor pulmonale, nephrotic syndrome, nephrosis, liver failure or cirrhosis, pulmonary edema, and thrombophlebitis or deep vein thrombosis in the arms); previous lymphedema treatment in either arm; uncontrolled intercurrent illness; psychiatric illness that would limit compliance with the study; and known allergy to electrode adhesives or compression fabrics | 239 | 1.47 | volume change in the at-risk arm ≥ 10% above the presurgical baseline | 10 | 0.03 |
| Soran et al (2016) | USA | Patients who underwent ALND for BC in LE monitoring program | Patients with preoperatively diagnosed clinical or subclinical LE and prior trauma on the operated upper limb | 180 | 1.76 | Girth difference of ≥2.0 cm in the involved limb versus the uninvolved limb. | 22 | 0.07 |
| Wetzig et al (2016) | Australia | Women with unifocal breast cancers <3 cm diameter and clinically negative lymph nodes | Not specified | 1088 | 5 | An increase of 15% or more from baseline in upper limb volume | 28 | 0.01 |
